# A Bayesian Model for Exploiting Application Constraints to Enable Unsupervised Training of a P300-based BCI

**DOI:** 10.1371/journal.pone.0033758

**Published:** 2012-04-04

**Authors:** Pieter-Jan Kindermans, David Verstraeten, Benjamin Schrauwen

**Affiliations:** Electronics and Information Systems, Ghent University, Ghent, Belgium; Cuban Neuroscience Center, Cuba

## Abstract

This work introduces a novel classifier for a P300-based speller, which, contrary to common methods, can be trained entirely unsupervisedly using an Expectation Maximization approach, eliminating the need for costly dataset collection or tedious calibration sessions. We use publicly available datasets for validation of our method and show that our unsupervised classifier performs competitively with supervised state-of-the-art spellers. Finally, we demonstrate the added value of our method in different experimental settings which reflect realistic usage situations of increasing difficulty and which would be difficult or impossible to tackle with existing supervised or adaptive methods.

## Introduction

A Brain-Computer Interface (BCI) [1], [2] is designed to allow direct communication between man and machine. In this work we focus on the P300 speller as presented by Farwell et al. [3] in 1988. This system allows people to spell words by looking at the desired character in a matrix shown on screen, thus enabling paralyzed patients to communicate with the outside world. The P300 speller has already been used by patients suffering from amytrophic lateral sclerosis [4], [5], and the study performed by Vaughan et al. [5] has shown that the spelling system is not limited to experiments in a laboratory but can be extended to home usage.

A common problem in all types of BCI's is the calibration procedure [6]. Brain activity differs substantially between people and between sessions. As a consequence, a BCI must be trained for a specific person before it can be used in practice. Most trainable methods require data for which the ground truth is known. Recording this data is very time consuming and a lot of effort has already been put into reducing the need for labeled data. The majority of the systems that require less data are based on adaptivity or the transfer of a classifier from one subject to another. To our knowledge, there exists no other method than the one proposed in this paper which is able to train a P300 classifier without any labeled data.

A second problem is the ground truth itself as discussed in [7]. In a BCI setup, the ground truth is often what the subject is expected to do, not what the subject does. An example: a subject can be confused and might sometimes focus on the wrong character during the P300 calibration procedure. A healthy subject using the P300 may detect his own mistake and simply say that he made a mistake. When this happens during the calibration procedure for a paralyzed user, there is no possible way to communicate about this and the classifier will be trained with wrongly labeled data. This can lead to severe problems and failure of the training.

This work tackles both these problems at once by proposing a completely unsupervised P300 speller. The unsupervised method allows us to do P300 spelling without calibration procedure or labeled data.

### The P300 Spelling Paradigm

**Figure 1:**
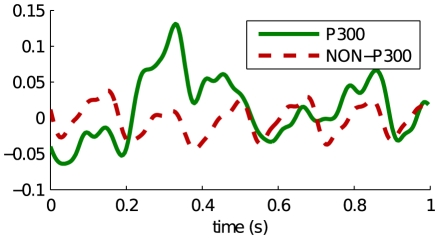
Plot showing the average P300 response versus the average background EEG.

The P300 wave is an Event Related Potential (ERP) elicited by a salient or attended stimulus [8]. It is a positive deflection which is typically detectable in the EEG measured around the parietal lobe, around 300 ms after the occurrence of the unexpected stimulus. It is based on the oddball paradigm, whereby a rare target stimulus is presented among common non-target stimuli. In Figure: 1, we have plotted the EEG for an averaged P300 response versus the average background EEG.

**Figure 2:**
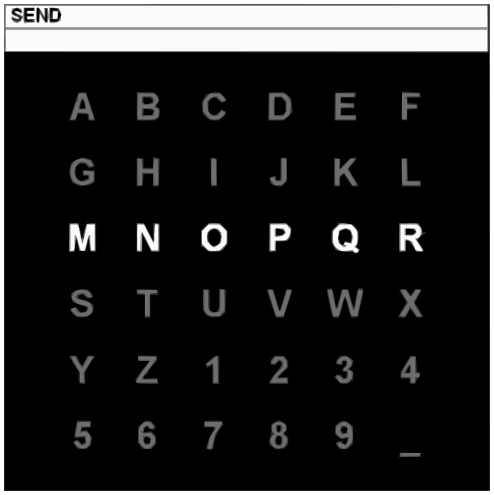
The speller matrix used in this work. Source: BCI Competition II dataset description.

In the case of a P300 speller [3], [9], the user is presented with a 6 by 6 grid of characters (shown in Figure 2) and focuses on the character he/she wants to spell. The rows and columns of the grid are highlighted in random order. When the desired character is highlighted, the subject sees an unexpected stimulus and a P300 wave is generated. By correlating the detection of the P300 wave and the (known) sequence of row/column highlights, the character which the user has focused on can be inferred.

Usually, due to the noisy nature and low spatial resolution of the EEG, a single series of highlights is not sufficient to detect the character with high accuracy. Therefore, several repeated highlightings (or epochs) of the same character are needed to achieve an accurate speller. Obviously, the number of repetitions needed for classification is an important characteristic of the speller, since this determines the effective spelling rate and, by extension, the usability of the system. As will become clear later, the use of these repetitions is a crucial reason why our unsupervised classifier works well.

Within the general framework of the P300 speller, several experimental settings can be discerned.

- Supervised vs. unsupervised: in the supervised case, the actual character being focused on by the user is known during training. For an unsupervised setting, this is not the case.
- Online vs. offline: in an offline setting, the whole testset is available in its entirety for classification. In the online setting, the characters are classified one by one and sequentially. The latter is most realistic and useful.
- Adaptive vs. non-adaptive: adaptive methods can fine-tune or adapt the trained model to new incoming data, whereas non-adaptive methods remain fixed during testing.

### Dataset

Reliably annotated and widely used P300 speller data is rare, so we used two datasets. The first dataset is a combination of two competition datasets with a limited number of subjects which is considered challenging, the other is an easier dataset with more subjects to show the robust applicability of the proposed method.

#### BCI Competition Datasets

We combined two datasets, namely from the BCI Competition II [10] and BCI Competition III [11], recorded by the Wadsworth Center at the New York State Department of Health. Both datasets are publicly available, and have been used in numerous previous BCI-related studies.

The full dataset consists of EEG recorded from three subjects (named A, B and C). Subjects A and B originate from BCI Competition III, subject C was used in BCI Competition II. The training data for subjects A and B contains 85 different characters, with 15 epochs per character. The test data is slightly bigger at 100 characters and 15 repetitions. For subject C, the training set consists of 42 characters and the test set contains 31 characters. For all subjects, 15 repetitions per character were recorded.

The EEG was recorded from 64 channels and digitized at a sampling rate of 240 Hz. The 6×6 spelling matrix (as shown in Figure 2) was shown for 2.5 s before the intensifications. An intensification of a row/column lasted 100 ms and the time between the intensifications is 75 ms.

Subject C can be considered an easy dataset: for the BCI competition II most participants were able to attain 100% spelling accuracy even with only 5 repetitions. Subjects A and B (from competition III) are considerably more challenging, as can be seen from the generally lower recognition rates, 72%–75% using 5 repetitions, compared to the perfect spelling results on subject C [10], [11].

#### Akimpech Datasets

In order to asses the performance of the proposed method on a wide range of subjects, we used the Akimpech P300 database [12]. We executed our experiments on data from 22 subjects. Each subject executed 4 spelling sessions. During the first session, they had to spell the words: CALOR, CARINO and SUSHI. In the second session they task was to spell SUSHI. The third and fourth session were free spelling sessions. The subject was able to choose the words. In the first three sessions, 15 intensifications per epoch were used. The fourth session used a subject specific number of iterations. Thus, we will only use the first three sessions to evaluate our method on. The first session is the training set. The second and third session combined form the test set. The training set contains 16 characters and the number of characters in the test set ranges from 17 to 29 depending on the subject.

In this dataset the EEG was recorded from 10 channels at 256 Hz. The same spelling matrix as in the BCI Competition dataset was used. This matrix was shown for 2 s before the intensifications. The stimulus duration was 62.5 ms and the time between the stimuli was 125 ms. There was an additional 2 s pause after the intensifications.

### Related Work

The P300 speller paradigm has been researched extensively. Topics in P300 research include but are not limited to: novel P300 speller representations, clinical tests on real patients and machine learning algorithms. We will restrict the in depth discussion of the related work to this last subfield.

The use of the BCI competition III dataset has been described in many BCI-related publications [7], [10], [11], [13]–[17], also after the competition closed. The following systems achieve the highest accuracies on BCI Competition III and will be used in a direct comparison:

- eSVM: Rakotomamonjy and Guige used an ensemble of Support Vector Machines (SVM) [17]. They divided the training set into 17 partitions, each partition containing EEG corresponding to 5 characters. Each partition was then used to do channel elimination and SVM training. The final classification was done by summing the outputs from the 17 resulting classifiers. The main advantage of this method is that it is still one of the best performing systems on that data set. The drawback is that the entire training procedure is rather cumbersome and that the classifiers are static.
- CNN-1 and MCNN-1: These methods, by Cecotti and Graser, are the currently one of the best performing classifiers [7]. They used Convolutional Neural Networks (CNN) to detect the P300. CNN-1 is trained on the entire data set, whereas MCNN-1 is an ensemble of CNN's. Each classifier in the ensemble is trained using a balanced data set by using all of the intensifications containing a P300 response and on only one fifth of the negative examples. Just as for the eSVM approach, the classifier outputs are summed to obtain the final prediction. Both CNN-1 and MCNN-1 produced excellent spelling predictions. However, like the eSVM approach, the training procedure is rather complex, computationally demanding and non-adaptive
- SUP: In [16] we presented a baseline method, trained using regularized class-reweighted regression [18]. The preprocessing is analogous to the procedure in our present work, with the exception of the band pass filter which was subject specific in the previous paper.
- OA-SUP is the adaptive version of SUP presented above. In OA-SUP, the adaptation procedure is kept as simple as possible:
  - Predict the next character
  - Label the individual intensifications based on the character prediction
  - Add the self labeled data to the training set
  - Retrain and repeat

This technique differs from other adaptive methods in the following manners. Firstly, we used the character selected by the P300 speller to label the data points instead of the classifier output on each sample. Secondly, the methods in [19]–[21] selected data points for the adaptation procedure based on a confidence score. The advantages of SUP and OA-SUP are the high spelling accuracy, the short training time and the simplicity of both the basic classifier and the classifier adaptation procedure. Furthermore the adaptation allows the OA-SUP method to produce excellent results with significantly reduced amounts of training data. However the adaptation scheme lacks theoretical foundations. The work presented in this paper can be seen as a mathematically sound and much more powerful extension of the OA-SUP system.

Some authors have described different adaptive P300 spellers. The most notable contributions are:

- Dähne et al. [22] have proposed an unsupervised LDA adaptation scheme. In LDA, the classifier weight vector is computed as
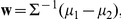
where 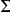 is the global covariance matrix, 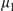 (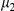) is the mean for class 1 (2). The global covariance matrix is computed without label information. Therefore it can also be updated without label information during spelling. This results in an adaptive classifier.

- Li et al. [21] have shown how Support Vector Machines (SVM) can be used in a self-training procedure to reduce the need for labeled data and how spelling accuracy can be improved trough adaptation.
- Panicker et al. [20] proposed adaptive methods based on Fisher Linear Discriminant (FLD) [23], [24] and Bayesian Linear Discriminant Analysis (BLDA) [25]. They evaluated self training and cotraining methods for the P300 speller. This method is related to our proposed method as BLDA is very similar to the classifier used in this work.
- An unsupervised method was proposed by Lu et al [19]. The underlying classifier was based on FLD. Although the proposed method allows P300 spelling without a subject specific calibration procedure, they still need labeled data to train the subject independent classification model. This subject independent model is then used as a starting point for the adaptive method. This differs from the work proposed in this paper where we have no need for labeled data at all.

Unfortunately, these methods are evaluated on data which is not publicly available which makes a comparison with our proposed method impossible.

The spelling results for the methods SUP, OA-SUP, eSVM, CNN-1 and MCNN-1 are given in Table 1, together with the results obtained by the methods proposed in this work.

**Table 1:** BCI Competition Spelling Accuracies.

	R	eSVM	CNN-1	MCNN-1	SUP	OA-SUP	OFF-US	OFF-US-T	ON-US-T	OA-US-T ÃŸ	RE-OA-US-T	OA-US
A	5	**72**	61	61	67	68	46.8 (4.0)	69.0 (0.0)	64.2 (0.9)	66.5 (0.5)	69.0 (0.0)	9.0 (7.4)
	10	83	86	82	88	**91**	89.4 (1.1)	**91.0** (0.0)	86.0 (0.0)	87.0 (0.0)	88.0 (0.0)	62.4 (4.1)
	15	**97**	**97**	**97**	96	95	95.8 (1.3)	96.0 (0.0)	94.0 (0.0)	96.0 (0.0)	96.0 (0.0)	86.6 (1.6)
B	5	75	79	77	**84**	**84**	76.3 (1.6)	79.0 (0.0)	75.0 (0.0)	75.0 (0.0)	79.0 (0.0)	53.0 (2.1)
	10	91	91	92	93	93	92.1 (1.3)	**95.0** (0.0)	91.0 (0.0)	94.0 (0.0)	95.0 (0.0)	87.9 (0.6)
	15	**96**	92	94	**96**	**96**	95.2 (0.6)	95.0 (0.0)	92.0 (0.0)	94.0 (0.0)	95.0 (0.0)	87.3 (1.1)
C	5	-	-	-	-	-	98.7 (1.7)	96.8 (0.0)	96.8 (0.0)	96.8 (0.0)	96.8 (0.0)	56.5 (5.5)
	10	-	-	-	-	-	100.0 (0.0)	100.0 (0.0)	100.0 (0.0)	100.0 (0.0)	100.0 (0.0)	83.5 (1.1)
	15	-	-	-	-	-	100.0 (0.0)	100.0 (0.0)	100.0 (0.0)	100.0 (0.0)	100.0 (0.0)	92.3 (1.7)

Percentage of correctly predicted characters. The first column indicates the subject, the second column the amount of repetitions per character. The values in braces are the standard deviation. Subject A and B are datasets from BCI Competition III. Subject C originates from Competition II. We have omitted a direct comparison with related work on subject C because different methods are able to achieve 100% on all experiments.

## Materials and Methods

### Preprocessing

Preprocessing is an essential part of a BCI system. However, this work focuses on the underlying classifier and not on the preprocessing. For this reason, and in order to reduce computational requirements, preprocessing is kept to a minimum and no subject specific parameters were used in our experiments.

The EEG is processed character by character, allowing the system to be applied online. The preprocessing consists of the following simple steps:

1. Application of a Common Average Reference filter [26].
2. Apply a bandpass filter with lower and upper cutoff frequencies of 0.5 Hz and 15 Hz respectively.
3. Normalization of each EEG channel to zero mean and unit variance.
4. Dimensionality reduction by subsampling the data by a factor 6. For each of the channels, we retain 10 samples per intensification. These samples are centered at the expected time step of the P300.
5. Addition of a bias term to the data.

These steps yield a 641 and a 101 dimensional feature vector for each intensification on the BCI Competition datasets and the Akimpech dataset respectively. Please note that the bias term is counted as a feature. The incorporation of this bias term into the preprocessing stage simplifies the discussion of the classifier used in this work (see later).

### The Basic Classifier

In this work we have employed a basic linear classifier which is closely related to Bayesian linear regression (BLR) [24], BLDA [24], [27] and FLD [23], [24]. The classifier is defined by the following assumptions:
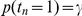


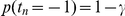


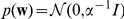


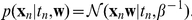
In this model, 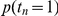 gives us the prior probability of a positive example, i.e. a P300 wave. The prior on the weight vector 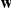 is a multivariate Gaussian distribution with zero mean and isotropic variance. This equals the assumption made by BLDA and the Bayesian formulation of ridge regression. The idea behind this prior is that we add regularization by keeping the weights in the weight vector small and as such the model complexity low.

**Figure 3:**
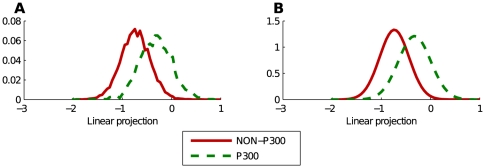
The projection of the EEG into one dimension produces two Gaussians. Figure A shows the histogram of the used EEG features projected into one dimension. Figure B shows two Gaussians fitted to this histogram. One Gaussian for the EEG containing the P300 response, one Gaussian for the data without P300 response. The vector 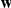 that was used in the projection was trained unsupervisedly on the data.

The conditional distribution on the EEG data 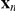 given the weight vector 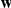 and the label 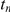 is a univariate Gaussian with variance 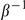. Please note that this is actually not a real distribution on 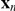 but using this interpretation allows the most straightforward derivation of the update equations and character predictions. This conditional distribution has the same functional form as the distribution on the target labels given the weight vector and the data in linear regression. However, there is a difference between linear regression and our model. Linear regression makes no assumptions about the input data 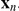 We assume that the data can be projected into one dimension where we obtain two Gaussians, one for each class, which share the same variance. This property may seem odd at first but it is less strict than Linear Discriminant Analysis, where the premise is that the data 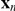 can be modeled by a mixture of multivariate Gaussians, where the 2 classes have separate means but share the same covariance matrix. The assumptions are also closely related to those made with FLD. In FLD we search for the projection that maximizes the separation of the class means and at the same time minimizes the class overlap. Furthermore Blankertz et al. [28] reported that in their experience ERP features are normally distributed. We point out that, since the resulting projection is a sum of random variables (100 or 640 in this work, depending on the dataset used), we can use the central limit theorem as a justification for assuming Gaussianity. We find that this assumption actually holds for our data, as illustrated in Figure 3. This figure shows class conditional histograms of the EEG data projected into one dimension along with Gaussians fitted to these histograms. The vector 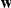, which is used in the projection, is the actual weight vector trained by our own unsupervised method.

When this classifier is used supervisedly, we need the posterior distribution on 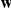 given the training data to make predictions about the class label:
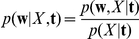


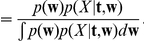
This posterior distribution is actually the same posterior distribution as the one obtained in BLR and BLDA. This is due to the fact the the prior on 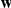 is the same and that 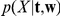 in our model has the same functional form as 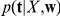 in the regression model. When we use a prior with zero mean 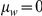 and isotropic variance 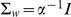 on 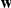 then the MAP estimate for 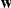 (or the mean of the posterior) is given by:
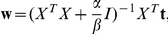
which equals the solution obtained by ridge regression, with the regularization constant 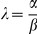. The advantage is that we have a closed form solution for 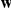.

The resulting conditional probability of a specific label given the data and the weight vector is:
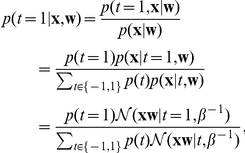
which is a binary distribution. If we look at it in function of 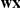, we see that it is a sigmoid function. In the case of 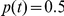 we see that 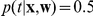 when 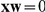. The prior can shift the point of equal probability towards the mean of less likely class. When the variance 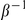 decreases then the sigmoid becomes steeper and the point of equal probability shifts towards 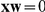.

A final and for this paper the most important advantage that we obtain using the classifier is the ability to model 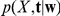. When we use 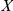 as observed variables, the class labels 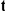 as latent variables and 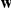 as the parameter to optimize then it is easy to see that we can apply the EM algorithm to it. This allows for unsupervised training of the weight vector 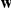. However, we will not use this model directly in the EM framework but a slightly more elaborate one with the P300 constraints embedded into it.

### The Expectation Maximization Framework

After the preprocessing we have the classification step. The classifier proposed in this work makes use of the Expectation Maximization (EM) [24], [29] framework, which we will first introduce in a generic manner before discussing the specifics of our classifier in the next section.

The EM framework is designed to find a maximum likelihood estimate for the parameter 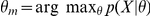 of a statistical model with observable data 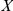 and latent variables 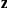. It is used when direct optimization of 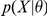 is difficult but where the joint distribution 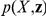 can be optimized more easily. In each step of the iterative algorithm a lower bound on 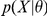 is optimized. The general algorithm can be described as follows:

1. Choose an initial parameter setting: 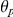

2. Perform the Expectation or E-step: for each assignment of the latent variables 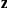 compute the conditional probability of 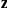 given the data 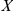 and the previous parameter 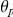


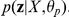

3. Perform the Maximization or M-step: find the most likely
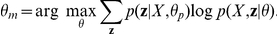

4. Let 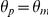, stop when the algorithm has converged to a solution, otherwise go to step 2

The convergence criterion can be defined in terms of changes to the parameter 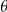 or as a function of the expected data log likelihood. The algorithm described above is the basic EM algorithm. In this work we will use both the basic algorithm and an extension with a prior distribution on 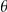. The addition of that prior changes the M-step to:
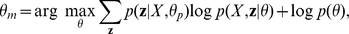
and we search for a Maximum A Posteriori (MAP) estimate of 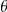 [24].

### Embedding the P300 Paradigm Directly into the Classifier

Before we can embed the P300 constraints into the model, we need to look at them in more detail. Assume that we have 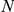 characters to predict, 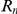 repetitions to predict character 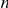 and 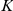 rows or columns, where 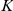 is in our case 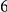. Let 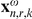 be the EEG for character 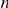 during repetition 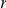 highlighting row or column 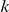, where 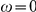 indicates that it is a row and 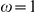 indicates that it is a column. Let 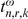 be the assigned label for the intensification, where a P300 intensification has label 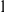 and a non-P300 intensification receives label 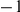. The P300 paradigm assumes that for each character, there is one column (row) that should generate a P300 response and this column (row) should be the same for all repetitions.To make the implications of this constraint clear, we would like to make an analogy. The detection of the row (or column) corresponding with the P300 response can be seen as a multiple choice exam. Assume that there are 15 questions in total and there are 6 different options for each question. If you add the constraint that for each question, always the same option contains the correct answer, then the exam becomes much easier.

The constraint can be embedded into the model by adding two indicator variables per character. These variables 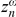 indicate which column (or row) should elicit a P300 response. The important part is that this indicator variable is shared among all rows or columns and all repetitions for a single character. The values that this variable can take are in 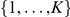 and these all receive the same probability mass: 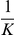. These indicator variables are integrated into the model by defining a conditional distribution on the label for a specific repetition 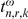 given the variable 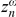. The complete model with constraints is:
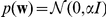


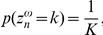


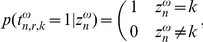


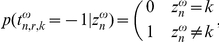


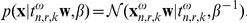
The superscript 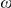 will be dropped from now on. The rows and columns are assumed to be independent, so we can actually treat the task as the classification of 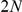 pseudo-symbols. How this model can be used in practice will be explained in the following sections, starting with the EM update equations.

### The Expectation Maximization Update Equations

We can fit the model directly into the EM framework. As we have already discussed, EM searches for a parameter 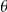 to optimally model our data 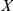. The parameter vector 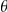 consist of 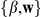 in the case of the P300 speller. The variable 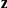 contains all the indicator variables for the entire test set and 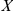 contains the preprocessed EEG. This gives us the following equation to optimize:
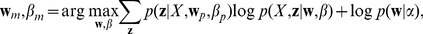
(1)where we have only used a prior on 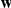 and not on 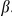 The optimal values for 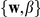 can be found by taking the derivative of Equation 1 with respect to that specific parameter, setting it equal to zero and solving the equation. The complete derivation for the update equations is available in the Document S1, we will only discuss the resulting equations.

The update equation for 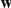 is the following:
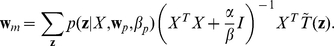
where 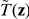 is the value that 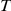, a vector containing the labels for the individual intensifications, can take for specific values of 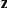. Formally this becomes: 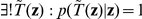. The updated weight vector is actually a sum over all possible ridge regression classifiers, weighted by the probability that the labels 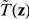, which were used in the training, are correct given 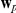. This brings us back to the standard supervised training procedure: if 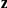 is given (we know the ground truth, the correct labels 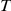) and we use the update equation, we obtain ridge regression.

The update for 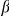 is as follows:
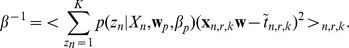
The updated value for 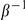 is again a weighted average. It is the mean squared error between the predicted (regression) output and the target labels, weighted by the probability that those target labels are correct given the current classifier.

The interaction between 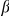 and 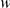 is easy to see when we consider a single assignment of target labels. For such a specific assignment 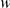 will be the weight vector which minimizes 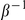. A small value for 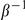 indicates that the class conditional Gaussian distributions are sharply peaked. This will in turn result in a high data log likelihood. Just as in FLD, we attempt to minimize the within class variance. A second important observation is that low values of 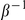 will lead to less regularization. This can be seen in the update equation for 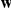 where 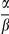 is the regularization parameter.

At this point we have only a single parameter left to tune: 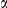. Setting 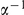 to 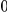 equals removing the prior from the model. This has the effect that the model is trained without regularization. This can be problematic for datasets where the underlying structure is hard to find. An example of such a hard dataset is subject A when only 5 repetitions are used. Setting 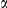 to a specific value gives a new meta parameter which has to be tuned. The preferable option is to optimize 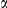 automatically. This can be done by optimizing Equation 1 with respect to 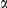. Doing so yields the following simple update equation:
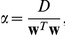
where 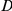 is the dimensionality of the weight vector 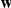. The behavior of the model is influenced by 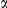 is as follows. The probability 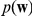 increases when 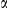 increases. A higher value for 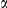 gives stronger regularization by forcing 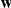 to use smaller weights. There is a caveat: a global optimum of the likelihood 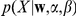 is reached when 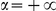. This optimum gives us a weight vector 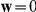, which is of course no real classifier. This implies that we expect our method to converge to a local optimum where 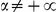. Although this may be counter-intuitive, we see a related problem in the training of Gaussian Mixture Models. Those models are also able to achieve an infinite likelihood, when one of the mixture components collapses on a single data point and the precision goes to infinity. The degenerate cases, for both the GMM as well as our model, are easily detectable and for our model, a simple solution could be to limit the range for 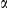. In practice, we find that when there is enough data available these problems seldom occur and there is no need to limit 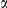, but in the online experiments where the available data is extremely limited, we will make use of a bound on 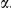


### From Classification to Spelling

In a P300 speller, we are not really interested in which intensifications generated the P300 response, what we really want to know is at which character the user was looking. For each character we compute the conditional probability for each row and each column that it contains the P300 given the data and the weight vector:
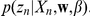
The desired row and desired column are simply the ones with the highest probability. The character at that coordinate in the letter matrix is the predicted character.

### Parameter Initialization and Classifier Selection

The initialization of the parameters is very simple in the default unsupervised and off-line case: 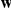 is drawn from 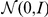, 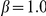 and 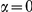. When we do this, we always initialize 2 spellers: one with the initial weight vector 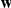 and one with 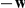.

**Figure 4:**
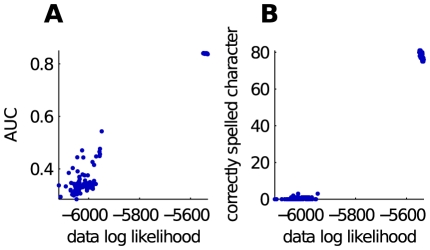
Scatter plots showing the quality of the classifier. Quality is measured in either AUC or characters predicted correctly versus the data log likelihood. The data used in this plot is created in the OFF-US experiment on subject B using 5 repetitions.

The idea behind this approach is that one of the classifiers will have an AUC slightly higher than chance level and the other slightly below. So the chance that one of them converges to a good classifier is high. When we have a pool of classifiers, we can use the data log likelihood as a criterion to find the best one. In Figure 4 we have made scatter plots where the performance of the classifier is given versus the data log likelihood. Performance is evaluated using the Area Under Curve in an Receiver Operator Curve on the individual intensifications and using the number of correctly predicted characters. A perfect binary classifier achieves an AUC of 1, a random classifier scores 0.5 and when the score is below chance level the labels are flipped. A total of 100 draws for 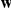 were used to generate these plots using the test data from subject B with 5 repetitions per character. There is a clear margin between the good classifiers and the ones with the flipped labels or random performance. Furthermore we can see that there is a strong link between AUC and spelling correctness. An important observation is that we find many classifiers which have very low AUC values. This means that we have to make sure that there are enough random initializations before we select a single classifier.

In this work, a single classifier will always be selected from a group of 10 initializations which gives us a total of 20 classifiers. For all experiments we repeat the initalization and classifier selection 10 times (i.e. we start with 10 groups of classifiers) to average over variations caused by the initialization.

## Results and Discussion

### Experiments

Our experiments are divided into two categories. The first two are completely off-line experiments. The last experiments are designed to emulate real world usage of our system.

#### OFF-US: Unsupervised Training on the Test Set

The first experiment consist of offline unsupervised training on the test set where 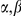 and 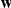 are all optimized. The use of the test set for training may appear confusing at first, but note that the training is done without ever using ground truth labels. This procedure was used to enable comparison with existing methods on the same data. Our goal with this setup was to find out what performance can be expected from our method when we pose no limits on the online applicability of our system. We used 10 groups to compute the mean of the results. In each group, we had 10 couples of classifiers, one initialized with 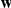 the other one with 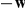 as described above. From each group we selected only one classifier, the one with the highest data log likelihood, to make the predictions, yielding 10 spellers for the final evaluation.

#### OFF-US-T: Increasing the Amount of Unlabeled Data

The second experiment was designed to show how the performance on the test set can be improved by increasing the amount unlabeled data from previous sessions. The setup is almost completely analogous to the first one, the only difference is that the unsupervised training algorithm now uses both the train and test set. The speller initialization and selection procedure is analogous to the experiment above.

#### ON-US-T: A Non-adaptive but Unsupervised Online System

The first online experiment uses a classifier OFF-US which is trained unsupervisedly on the train set and tested on unseen data from the same subject. In these experiments we used 15 repetitions per character to do the initial training. Training was done using 10 groups of classifiers. After the training of the classifiers, we chose 1 classifier from each group based on the data log likelihood obtained on the train set. These classifiers were then evaluated on the test set. The importance of this experiment lies in the fact that creating the unlabeled data is easy. One can then simply use the speller. When we are able to use the unseen data to build an (initial) classifier with good performance we can start spelling without a new calibration procedure.

#### OA-US-T: Improving the Online Spelling Trough Adaptation

Our second experiment re-uses the classifiers from the ON-US-T experiment. This online experiment is representative of repeated usage of the speller. A previous session, from which we have no knowledge about the spelled words, is used to initialize the system. To increase performance we adapt the system to the new sessions. During the evaluation of the system on the test set, the classifier receives the EEG for the character that it has to predict. It adds the new data to the unsupervised training set. Now the training data has grown, and it updates the classifier with 3 EM iterations. This adapted classifier is then used to predict the character. This procedure is then repeated iteratively until the entire test set is processed and added to the training data.

For completeness we also did an evaluation of classifiers after the entire test set was processed. These classifiers are called RE-OA-US-T and these produce results which are not representative of an online experiment but can shows us whether the classifier has improved or not.

#### OA-US: The True Challenge, Spelling Without any Prior Knowledge

This final experiment is the most challenging. The classifier starts initially untrained, without any data. To predict a character we add the EEG for that character to the training set and perform 3 EM iterations. After the EM procedure we predict the character. Then we go to the next one, each character increasing the unsupervised training set.

This experiments needs a special initialization procedure. The low amounts of data that are available at the start of the experiment allow for over-fitting or make the probability of for the pathological value for 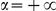 very high. To counter this we constraint 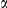 to a maximum of 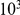. In each group of spellers we draw 10 values for 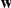. Each draw results in a couple of spellers, initialized with 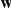, one with 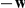 just as before. For each character, each couple of spellers does 3 EM iterations. To predict a character we select the speller from the group with the highest data log likelihood. This is completely analogous to the previous training procedures. After a character is predicted we evaluate the data log likelihood for all the seen data. From each couple of speller we select the one with the highest data log likelihood. The other speller in the couple is then reinitialized. It receives the values for 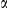, 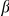 from the other classifier. The weight vector is reset by selecting 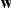 from the one with the best data log likelihood and resetting the bad weight vector to 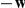. This procedure makes sure that the different classifiers within a couple start each EM iteration with opposite labels. This adapted procedure is designed to counter the effects from bad initializations and classifiers which become very similar.

### Evaluation

Before we start the analysis of the different experiments we will give some information about the error measures used in this work. We will use both the spelling and the Area Under Curve (AUC) as an error measure.

The spelling accuracy is essential to evaluate a P300 speller as it works directly on the task at hand. Nevertheless, it is not really a stable measure. Most systems, including ours, classify the individual intensifications and combine the outputs to predict a character. Two classifiers can make the same amount of mistakes on the individual intensifications and therefore the two classifier are of equal quality. When the classifiers make their mistakes on specific intensifications, it can result in a difference in spelling prediction. Therefore we propose to use the AUC as a measure of classifier quality. The AUC is the area under the Receiver Operator Curve (ROC). In such an ROC, the False Positive Rate is plotted versus the True Positive Rate for different classifier thresholds. An AUC of 1 indicates perfect classification, 0.5 is the score obtained by a random classifier and a score below 0.5 means that we have a classifier which has swapped the labels. We argue that this is a more relevant error measure than looking at the precision or recall of the classifier. The AUC compares the classifier outputs for positive and negative examples relative to each other. Just as the speller compares the outputs for each of the rows and columns and selects the best one. Furthermore we see in Figure 4 that there is a high correlation between AUC and spelling performance.

### Discussion

In our discussion we will address the experiments in the order they were introduced above, starting with the off-line experiments and finishing with the online ones. The spelling results for the BCI Competition experiments are given in Table 1. Where applicable we have given the mean and standard deviation for 10 different spellers. The results for the Akimpech dataset are available in Table 2. We have averaged out the means over all subjects and the standard deviation over the subject means is given. The table containing the individual results and standard deviations over experiments is given in the Document S2.

**Table 2:** Akimpech Spelling Accuracies.

R	OFF-US	OFF-US-T	ON-US-T	OA-US-T	RE-OA-US-T	OA-US
5	87.6 (11.9)	88.3 (11.9)	86.8 (13.0)	87.9 (12.2)	88.6 (11.5)	61.2 (25.8)
10	96.9 (5.2)	96.5 (5.2)	95.6 (5.9)	96.9 (4.5)	96.7 (5.2)	85.6 (14.6)
15	98.8 (2.8)	98.8 (2.8)	97.8 (3.7)	98.3 (3.4)	98.8 (2.8)	93.1 (6.0)

Percentage of correctly predicted characters averaged out over subjects from the Akimpech dataset. The first column indicates number of of repetitions per character. The values in braces are the standard deviation computed over the means of the different subjects. Subject specific results can be found in the supplementary material.

#### Off-line Experiments

To start, we will compare OFF-US, which is trained on the unlabeled test set with eSVM, which is the winner of BCI Competition III and the method to beat on this dataset. The eSVM approach scores 97% on subject A and 96% on subject B. Our unsupervised approach achieves an average accuracy of 95.8% on A and 95.2% on B and thus performs slightly lower. The results for the individual classifiers which were used to compute the final results, range from 95% to 98% on subject A and from 95% to 97% on subject B. Furthermore the low variance on the accuracy indicates once more that our training procedure and classifier selection is a reliable method: using the data log-likelihood to select the classifiers seems to be a valid approach. The results on the significantly less challenging data from subject C are 100% correct for 10 and 15 repetitions. When we reduce it to 5 repetitions, we achieve 98.7%. Some classifiers spelled 1 character wrong (actually only one row or column), the others made no mistakes. Using only 10 repetitions we obtain very high scores on subjects A and B. The results are comparable to those obtained with eSVM and the CNN's but our own adaptive subject specific and supervised classifier performs better on subject B. The result for subject A with only 5 repetitions is very poor while those on subject B are comparable to the other methods. This is actually a very important result. First of all, it shows the limitations of our method. Subject A is very challenging and most methods have difficulties on this data with a low number of repetitions. To find out how the data is structured, our method uses the constraints posed by the paradigm. The lower the number of epochs, the less information can be inferred from these constraints, and thus the harder the task. The consequence is that due to the lack of constraints the application of EM to the basic classifier (without the application constraints) will result in poor performance. We would also like to point out that the number of characters used in the unsupervised training influences the performance, we will defer the analysis of the influence of the amount of data to the next experiments.

The average accuracy obtained on the Akimpech dataset with the standard SWLDA classifier in BCI2000 is 98.1% for 15 epochs. These SWLDA results are included in the subject specific description of the dataset. Only exact figures are available for 15 epochs. Our proposed method achieves an average accuracy of 98.8%. When we examine the subject specific results, which are given in the supplementary material, we discover that on 18 out of 22 subject we are able to spell the entire test set correctly. The SWLDA classifier is able to achieve this for 16 subjects. After reducing the number of epochs to 10, we get an accuracy of 96.9. Spelling is still perfect for 15 subjects. A total of 5 subjects is able to do this for 5 epochs. The average result is 87.6% in this case.

**Figure 5:**
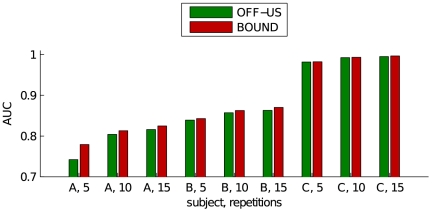
Bar graph showing the performance, measured in AUC, on the test. The classifier OFF-US is trained unsupervisedly on the test set. The BOUND is trained supervisedly on the test set without regularization.

A question one can ask is: how does this classifier compare to one which is trained supervisedly on the same data? To investigate this, we have trained a classifier using the proposed EM scheme where the probabilities of a column or row were fixed such that they reflect the ground truth. As we want the the best possible classifier for this data, we have omitted the optimization of 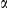 and we fixed it to zero, effectively removing regularization. This is not a problem in the current experiment as we will evaluate on the same set. We do not consider the generalization properties of the proposed method in this experiment. For this reason, over-training will give us the most challenging bound possible. As the resulting classifier predicted nearly all of the characters correctly, we will not use the spelling as performance measure but we will look at the AUC instead. We evaluated the AUC on the entire test set, even when the number of repetitions was reduced. We made this choice to be able to compare all the classifiers on the same data. Because only the classifiers trained with 15 epochs have processed the entire test set we obtain slightly biased results. The results themselves are shown in Figure 5. It is clear that we can get very close to the supervised system on the less challenging setups. Also, the resulting classifier on A using 5 repetitions is still significantly better than random. This experiment confirms that we are able to train the classifier on unlabeled data.

After our initial experiment we wanted to investigate how the performance is influenced by the availability of more unlabeled data. The OFF-US-T classifier is again trained completely unsupervisedly, but now the entire (unlabeled) training set is combined with the test set to train the classifier. A global observation is that due to the increase in data, the variance on the character prediction has vanished. In most cases we see an increase in performance and we will discuss only the most salient results. On subject A we achieved 69%. This is an increase of 22.2% over the previous experiment. We outperformed the CNN's and came significantly closer to the 72% obtained by the much more complex and supervised eSVM method. Performance on subject B (79%) when we are limited to 5 repetitions is as good as the other methods. Increasing the number of repetitions to 10 results in an accuracy of 95%. This is an improvement over all the compared methods. As the performance on subject C is still excellent, there is not much to discuss. The drawback of adding training data is that the positive outliers on 15 epochs from the previous experiment have disappeared. The Akimpech dataset exhibits similar behavior. We observe a slight increase in spelling accuracy for 5 epochs, a minor decrease for 10 epochs and equal performance for 15 epochs.

There is another advantage which cannot be seen from the table or the plots. The number of initializations which produce spellers with low quality is considerably lower when compared to the previous experiment. This brings us to an important practical aspect: when our proposed method is used, it is beneficial to use (unlabeled) data from previous experiments. By using the speller as a communication tool (i.e. freely spelling what you want, not what you have to spell in order to create a labeled data set) we can record data which will improve the speller itself. Giving exact figures about how many initializations are necessary is not feasible. We found that with 10 initializations we can achieve very stable results on all the datasets. By analyzing the variance of the individual experiments we see that there is almost never any variance on the spelling performance. As a result we can conclude that increasing the number of initializations will not increase the maximum attainable performance level but will increase the probability of achieving the maximum performance possible.

#### Online Experiments

Although the previous experiments have been very informative, they are limited in the sense that they do not predict the performance for practical usage. To do this, we need experiments which simulate online usage. We did not perform any realtime experiment but we did mimic the circumstances of an online setting. We feel that using the publicly available data in an online setup produces results which are scientifically the most valuable as other people can compare with them.

In order for the experiments to be truly representative for a real application, each step of EEG processing has to be possible online. This is the case in our system: there are no subject specific parameters which have to be selected by cross validation and the EEG is preprocessed on a character by character basis.

First, we will focus on the computational requirements of the proposed methods. Afterwards we will discuss the spelling performance.

In the first online experiment, ON-US-T there is no difference in efficiency between our classifier and the standard BLDA approach. The different initializations do not induce an extra computational penalty as the ON-US-T classifiers are trained and selected offline, i.e. before the spelling sessions. The result of this approach is that only a single classifier is needed for each evaluation.

The classifiers from the ON-US-T experiments are used to initialize the OA-US-T experiments. The classifiers are updated online as more and more unlabeled EEG is added to the unlabeled training set. Using the ON-US-T classifiers as initialization, gives us the benefit that only one classifier has to be updated. The time needed to perform a single EM iteration (i.e. classifier update) scales linearly with both the number of characters and the number of epochs. Experiments on the Akimpech (BCI Competition) dataset have shown that the EM update takes 0.85 ms (3 ms) per epoch. When 15 epochs are used per character, then we can execute 3 EM iterations within 2 seconds for up to 52 (14) characters on the Akimpech (BCI Competition) data. All of these results are obtained using a non-optimized python implementation on a standard laptop.

The OA-US experiment has the same computational requirements for the EM updates. The classifier selection and spelling of the new character is almost instantaneous and as such negligible. In contrast to the previous experiment, the OA-US needs different initializations per test run in order to achieve stable performance. Especially on the BCI Competition datasets. The updates for the individual classifiers can be done in parallel and they will not increase the spelling given enough computational power.

We are mainly interested in the behavior of the classifiers. We did not take the time needed to compute the updates into consideration. Nevertheless, when there are timing restrictions posed on the classifier updates – Which is the case in a real online experiment – we suggest the following approach. If the updates take up to much time then one should first spell the character before updating the classifier. These updates can be computed during the intensifications for selecting the next character. A single epoch takes 2.25 s (2.1 s) in the Akimpech (BCI Competition) database. Using this approach, 882 (250) characters can be analyzed during 3 EM iterations on the Akimpech (BCI Competition). This number is independent of the number of epochs because both the time needed for the update and the time needed for the intensifications scales linearly with the number of epochs.

Our first and least complex online setup: ON-US-T is a speller which is trained unsupervisedly on the entire train set, for the full 15 epochs. A total of 10 classifiers were selected. Each classifier came from a different group, with in each group 10 initial draws for 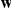 and 2 initialization per 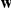. The selection criterion was the data log likelihood on the train set. This means that the test set was not used before evaluation. Overall the results show us that our method performs in a satisfactory manner. Once more we obtained near perfect figures on subject C. The experiment with 15 repetitions shows a slight decrease in performance compared to our offline experiments and the supervised methods. The results obtained with 10 repetitions are comparable to the supervised techniques. Only subject A with 5 repetitions performs quite poorly (64.2%), but this result is still better than the CNN's. The variance on the results is also extremely low, 0.9% for subject A using 5 repetitions and 0.0% elsewhere. This indicates once more that the selection criterion is very stable. In the analysis of the Akimpech dataset we notice the same effects: the performance is slightly lower than both offline experiments with accuracies of 86.8%, 95.6% and 97.8% for 5, 10 and 15 repetitions. All in all the results show that we can build a classifier which is trained without knowing the ground truth and which can readily be used in a new session. In addition, this approach introduces no computational penalty over other classifiers during online spelling.

**Figure 6:**
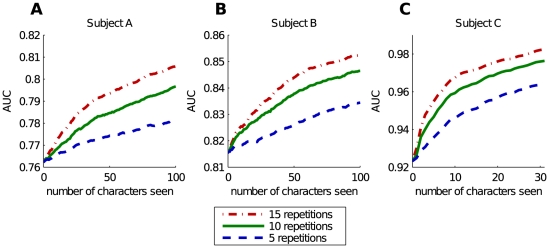
Classifier improvement trough adaptation. The initial classifier was trained unsupervisedly on the train set with 5 repetitions. The classifier was adapted to the EEG by feeding it the EEG character by character and performing EM on the original training set combined with the new EEG.

Our second online experiment, OA-US-T, is focused on adaptation. The results given in Table 1 show a slight increase in accuracy compared to the non-adaptive method, with the Akimpech dataset corroborating these results once more. For completeness we also re-evaluated the classifier which we obtained after processing the entire test set. The results for this off-line re-evaluation can be found under RE-OA-US-T. The performance of the final classifier is very close to our best performing setup: the classifier OFF-US-T which was trained offline on both the train and test data. This proves that the adaptation works and consistently improves the classifier. This is confirmed by the traces of the AUC on the test set. Figure 6 shows the evolution of the AUC in function of the number of processed character. As we start from the ON-US-T classifiers, the setups for 5, 10 and 15 repetitions begin at the same point. We see that with almost each new character, the performance increases. It is also clear that an increased number of epochs has a positive effect on the performance.

**Figure 7:**
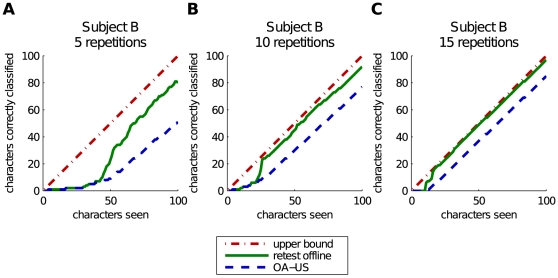
Plots showing the performance obtained by 3 single online initializations on subject B, each using a different number of repetitions to predict a character. The horizontal axis represents the number of characters processed. The vertical axis represents how many of these characters were predicted correctly. The dashed line shows us how many characters the online classifier has predicted correctly (starting with an initially untrained classifier). The solid line shows how many characters the current classifier can predict correctly if we re-test it on all of the previously processed characters. The dash-dot line represents the upper bound on the performance which equals the number of characters seen.

The adaptive classifier was initialized with a large chunk of unlabeled data. In our final experiment we omitted the data from the previous session. We perform online spelling with an initially completely untrained classifier. The data gets added iteratively as we process more and more characters. This method is called OA-US and the results are given in the final column of Table 1. When we use 15 repetitions, we obtain 86.6%, 87.3% and 92.3% on respectively A, B and C. A classifier which is trained with data from previous experiments will produce better results. As we decreased the number of repetitions to 10 the performance on subject A became poor: only 62.4% was correctly predicted. In the most complex case, 5 repetitions per character, our method fails completely on subject A. On average only 9% correctly classified, which is still above chance level (2.78%). The results on B (53.0%) and C (56.5%) are significantly better. Results an the Akimpech data are similar with 61.2%, 85.6% and 93.1% for 5, 10 and 15 epochs. The individual spelling accuracies on this dataset and 5 repetitions range from 23.1% to 93.5%. By increasing the number of repetitions to 15, the lowest accuracy is achieved on subject GCE with 75.3% and spelling is perfect for subject ASR. When only a single initialization is used per experiment, the performance on the Akimpech dataset drops to 57.8% for 5, 76.1% for 10 and 81.9% for 15 epochs. The reason for the failure on specific subjects when using 5 epochs is simple. These classifiers have to learn on the fly. As we have already shown, our classifiers benefit from an increase in data and epochs. We have illustrated these effect in Figure 7. In these plots we have drawn traces of the online experiment for a single initialization on subject B for 5, 10 and 15 repetitions. The horizontal axis represents the number of characters already predicted. The vertical axis represents the amount of characters predicted correctly. The dash-dot line is an upper bound on the performance, simply the number of characters already seen. The dashed line shows the online performance of our classifier. This line represents the performance during practical usage. The solid line shows the number of characters speller correctly when the classifier is re-evaluated on all previously seen characters, which can be compared to US-OFF performance.

These figures clearly show that our classifier needs to observe a number of characters before it will start to work. The harder the dataset, the more data is needed. The results in Table: 1 confirm this. We obtain the highest scores on the easy data from C and the lowest on A. Furthermore we see that the more repetitions, the less data is needed for the classifier to work. For 15 repetitions, the resulting classifier obtains results which are very close to perfect.

A second observation is that our classifier has a non-linear “eureka” transition when it receives enough data. When this happens, the classifier quality increases quickly and it corrects the mistakes it made previously. This can be seen by the solid line, representing the accuracy of the current classifier on all previously seen characters, which rises rapidly during the transition and then stays at a more or less fixed distance from the upper bound. From this point on, a wrongly predicted character becomes rather rare. This is represented in the plots by the online prediction line which runs parallel to the bound.

All in all we can conclude that our online classifier without prior knowledge produces good results. However, one should keep in mind that we need a warm up period to generate enough data to allow the classifier to be trained unsupervisedly. In short: we have removed the need for labeled training data but not the need for data. We argue that this does not limit the usability of the proposed method. Current systems have to rely on a supervised calibration procedure. Furthermore subject A and B are notoriously hard P300 datasets. The results obtained on subject C indicate that for patients with a clean P300 wave, our method needs only a tiny amount of unlabeled data when 10 or 15 epochs are used. On the Akimpech dataset, we have observed the same behaviour.

### Conclusion and Future Work

In this work we proposed a P300-based speller which has a linear classification backend and is computationally undemanding. Moreover, the classifier is trained in an unsupervised manner, yet it still is able to rival the performance of the more complex and supervised state of the art methods. We evaluated our method in several experimental settings, in either an offline setting in order to assess the performance w.r.t. upper bounds on the performance, or in an online setting to closely mimick realistic circumstances.

We feel that this method can form the basis for additional research into building a robust and practically usable P300 speller. Future work should focus on different directions, which are complementary. A first interesting topic is how we can improve the performance by using subject transfer. A second promising option is to incorporate a language model directly into the EM update scheme. Both the subject transfer and the language model should enable an improvement of the spelling accuracy and a reduction of the warm-up period for the online experiment without prior subject specific knowledge.

Finally, we have shown that the constraints posed by the basic paradigm can form the basis for unsupervised training. This implies that our approach should be transferable to the wide range of alternative P300 spelling setups available.

## Supporting Information

Document S1 **Derivations of the Update Equations.** This file contains the full derivation of the update equations.
(PDF)

Document S2 **Subject Specific Results on the Akimpech Dataset.** This file contains the subject specific results for all experiments performed on the Akimpech Dataset.
(PDF)
