# Supplementary material for: A Bayesian Model for Exploiting Application Constraints to Enable Unsupervised Training of a P300-based BCI
Source: PLoS One. 2012 Apr 4;7(4):e33758. doi: 10.1371/journal.pone.0033758 (PMC3319551; doi:10.1371/journal.pone.0033758)
Supplement: Document S1 — Derivations of the Update Equations. This file contains the full derivation of the update equations. (PDF) [file pone.0033758.s001.pdf]

S1: Derivations of the Update Equations Supplementary Material for A Bayesian Model for Exploiting Application Constraints to Enable Unsupervised Training of a P300 based BCI

1 The proposed model

In the paper we have proposed the following model:

$$
\begin{aligned}
p(\mathbf{w}) &= \mathcal{N}(\mu_w, \Sigma_w) \\
p(z_n^\omega = k) &= \frac{1}{K}, \\
p(t_{n,r,k}^\omega = 1 | z_n^\omega) &= \begin{cases} 1 & z_n^\omega = k \\ 0 & z_n^\omega \neq k \end{cases}, \\
p(t_{n,r,k}^\omega = -1 | z_n^\omega) &= \begin{cases} 0 & z_n^\omega = k \\ 1 & z_n^\omega \neq k \end{cases}, \\
p(\mathbf{x} | t_{n,r,k}^\omega \mathbf{w}, \beta \quad) &= \mathcal{N}(\mathbf{x}_{n,r,k}^\omega \mathbf{w} | t_{n,r,k}^\omega, \beta^{-1}).
\end{aligned}
$$

In order to simplify the equations, we will drop the superscript ω and use $2N$ pseudo-labels instead.

2 Derivations of update Equations for EM

We want to find update equations for $\mathbf{w}, \beta$ (and α) that maximizes:

$$\sum_{\mathbf{z}} p(\mathbf{z}|X, \mathbf{w}_p, \beta_p) \log p(X, \mathbf{z}|\mathbf{w}, \beta) + \log p(\mathbf{w}|\mu_w, \Sigma_w), \tag{1}$$

where the full model from above is used. To optimize for a specific parameter, we take the derivative with respect to that parameter, set the resulting equation

equal to zero and solve for that parameter. But to keep the discussion of the update equations readable, we have to simplify:

$$\sum_{\mathbf{z}} p(\mathbf{z}|X, \mathbf{w}_p, \beta_p) \log p(X, \mathbf{z}|\mathbf{w}, \beta) \tag{2}$$

into something which is simpler to use in the derivation. We will start by using the following notation: $\theta_p = \{\beta_p, \mathbf{w}_p\}$ and $\theta = \{\beta, \mathbf{w}\}$ which reduces Equation 2 to:

$$\sum_{\mathbf{z}} p(\mathbf{z}|X, \theta_p) \log p(X, \mathbf{z}|\theta) \tag{3}$$

We continue by expanding Equation: 3 by making use of the fact that $p(\mathbf{z}|X, \theta_p)$ and $p(X, \mathbf{z}|\theta)$ are both a product over all rows and columns giving us a product over $2N$ different indicator variables:

$$\begin{aligned}
& \sum_{\mathbf{z}} p(\mathbf{z}|X, \theta_p) \log p(X, \mathbf{z}|\theta) \\
= & \sum_{z_1} \sum_{z_2} \cdots \sum_{z_{2N}} \prod_{n=1}^{2N} p(z_n|X_n, \theta_p) \sum_{m=1}^{2N} \log p(X_m, z_m|\theta) \\
= & \sum_{z_1} p(z_1|X_1, \theta_p) \sum_{z_2} p(z_2|X_2, \theta_p) \ldots \sum_{z_{2N}} p(z_{2N}|X_{2N}, \theta_p) \sum_{m=1}^{2N} \log p(X_m, z_m|\theta) \\
= & \sum_{z_1} p(z_1|X_1, \theta_p) \log p(X_1, z_1|\theta) \\
+ & \sum_{z_1} p(z_1|X_1, \theta_p) \sum_{z_2} p(z_2|X_2, \theta_p) \ldots \sum_{z_{2N}} p(z_{2N}|X_{2N}, \theta_p) \sum_{m=2}^{2N} \log p(X_m, z_m|\theta) \\
= & \sum_{n=1}^{2N} \sum_{z_n} p(z_n|X_n, \theta_p) \log p(X_n, z_n|\theta).
\end{aligned}$$

This sum of sums makes the derivations of Equation: 2 with respect to $\mathbf{w}$ and β much less complicated because only:

$$p(X_n, z_n|\theta) = p(X_n, z_n|\mathbf{w}, \beta) \tag{4}$$

depends on $\mathbf{w}$ and β.

Analyzing Equation: 4 allows us to further simplify the optimization process:

$$\begin{aligned}
& p(X_n, z_n|\mathbf{w}, \beta) \\
= \; & p(z_n) \sum_{T_n} p(T_n|z_n) p(X_n|T_n, \mathbf{w}, \beta) \\
= \; & p(z_n) p(X_n|\tilde{T}_n(z_n), \mathbf{w}, \beta),
\end{aligned}$$

where T_n are the labels assigned to the individual EEG sequences. Due to the distribution on $t_{n,e,k}|z_{n,e,k}$ there exists one unique assignment for T_n which has probability mass 1. We will indicate this assignment by $\tilde{T}_n(z_n)$. All the other assignments have zero probability mass. This result can be plugged into Equation: 2:

$$\begin{aligned}
&\sum_{\mathbf{z}} p(\mathbf{z}|X, \mathbf{w}_p, \beta_p) \log p(X, \mathbf{z}|\mathbf{w}, \beta) \\
= &\sum_{n=1}^{2N} \sum_{z_n=1}^{K} p(z_n|X_n, \mathbf{w}_p, \beta_p) \log p(X_n|\tilde{T}_n(z_n), \mathbf{w}, \beta) \\
+ &\sum_{n=1}^{2N} \sum_{z_n=1}^{K} p(z_n|X_n, \mathbf{w}_p, \beta_p) \log p(z_n),
\end{aligned}$$

from we see that only the first term in the sum depends on $\mathbf{w}$ and β. This means that when we take the derivative of Equation: 2 with respect to β or $\mathbf{w}$ the second part vanishes and only the derivative of:

$$\begin{aligned}
&\sum_{\mathbf{z}} p(\mathbf{z}|X, \mathbf{w}_p, \beta_p) \log p(X, \mathbf{z}|\mathbf{w}, \beta) \\
= &\sum_{n=1}^{2N} \sum_{z_n=1}^{K} p(z_n|X_n, \mathbf{w}_p, \beta_p) \log p(X_n|\tilde{T}_n(z_n), \mathbf{w}, \beta) \qquad (5)
\end{aligned}$$

has to be found.

2.1 The update equation for w:

The previous section allows us to quickly find the update equation for $\mathbf{w}$. The derivative of Equation: 1 with respect to $\mathbf{w}$ is:

$$\begin{aligned}
&\frac{d}{d\mathbf{w}} \sum_{\mathbf{z}} p(\mathbf{z}|X, \mathbf{w}_p, \beta_p) \log p(X, \mathbf{z}|\mathbf{w}, \beta) + \frac{d}{d\mathbf{w}} \log p(\mathbf{w}|\mu_w, \Sigma_w) \\
= &\sum_{n=1}^{2N} \sum_{z_n=1}^{K} p(z_n|X_n, \mathbf{w}_p, \beta_p) \frac{d}{d\mathbf{w}} \log p(X_n|\tilde{T}_n(z_n), \mathbf{w}, \beta) + \frac{d}{d\mathbf{w}} \log p(\mathbf{w}|\mu_w, \Sigma_w),
\end{aligned}$$

where we have used the result from Equation: 5 to simplify the first term.

The derivation of $\log p(X_n|\tilde{T}_n, \mathbf{w}, \beta)$ with respect to $\mathbf{w}$ can be found as follows:

$$
\begin{aligned}
& \frac{d}{d\mathbf{w}} \log p(X_n|z_n, \mathbf{w}, \beta) \\
= \ & \sum_{r=1}^{R_n} \sum_{k=1}^{K} \frac{d}{d\mathbf{w}} \log \mathcal{N}(\mathbf{x}_{n,r,k}\mathbf{w}|\tilde{t}_{n,r,k}(z_n), \beta^{-1}) \\
= \ & \sum_{r=1}^{R_n} \sum_{k=1}^{K} \frac{d}{d\mathbf{w}} \left[-\frac{1}{2}\beta(\mathbf{x}_{n,r,k}\mathbf{w} - \tilde{t}_{n,r,k}(z_n))^2 \right] \\
= \ & \sum_{r=1}^{R_n} \sum_{k=1}^{K} \left[-\beta(\mathbf{x}_{n,r,k}\mathbf{w} - \tilde{t}_{n,r,k}(z_n))\mathbf{x}_{n,r,k}^T \right] \\
= \ & -\beta \left(X_n^T X_n \mathbf{w} - X_n^T \tilde{T}_n(z_n) \right).
\end{aligned}
$$

The value for $\frac{d}{d\mathbf{w}} \log p(\mathbf{w}|\mu_w, \Sigma_w)$ is the following:

$$
\begin{aligned}
& \frac{d}{d\mathbf{w}} \log \mathcal{N}(\mu_w, \Sigma_w) \\
& = \frac{d}{d\mathbf{w}} - \frac{1}{2}(\mathbf{w} - \mu_w)^T \Sigma_w^{-1} (\mathbf{w} - \mu_w) \\
& = -\Sigma_w^{-1}(\mathbf{w} - \mu_w).
\end{aligned}
$$

We can use these results and plug them into the equation above:

$$
\begin{aligned}
& = & & -\sum_{n=1}^{2N} \sum_{z_n=1}^{K} p(z_n|X_n, \mathbf{w}_p, \beta_p)\beta \left(X_n^T X_n \mathbf{w} - X_n^T \tilde{T}_n(z_n) \right) - \Sigma_w^{-1}(\mathbf{w} - \mu_w) \\
& = & & \sum_{n=1}^{2N} \sum_{z_n=1}^{K} p(z_n|X_n, \mathbf{w}_p, \beta_p)\beta \left(X_n^T X_n \mathbf{w} - X_n^T \tilde{T}_n(z_n) \right) + \Sigma_w^{-1}\mathbf{w} - \Sigma_w^{-1}\mu_w) \\
& = & & \sum_{n=1}^{2N} \sum_{z_n=1}^{K} p(z_n|X_n, \mathbf{w}_p, \beta_p)\beta \left(X_n^T X_n + \frac{\Sigma_w^{-1}}{\beta} \right) \mathbf{w} \\
& & & -\sum_{n=1}^{2N} \sum_{z_n=1}^{K} p(z_n|X_n, \mathbf{w}_p, \beta_p)\beta \left(X_n^T \tilde{T}_n(z_n) \right) - \Sigma_w^{-1}\mu_w \\
\beta \left(X^T X + \frac{\Sigma_w^{-1}}{\beta} \right) \mathbf{w} & = & & \sum_{n=1}^{2N} \sum_{z_n=1}^{K} p(z_n|X_n, \mathbf{w}_p, \beta_p)\beta \left(X_n^T \tilde{T}_n(z_n) \right) + \Sigma_w^{-1}\mu_w \\
\left(X^T X + \frac{\Sigma_w^{-1}}{\beta} \right) \mathbf{w} & = & & \sum_{n=1}^{2N} \sum_{z_n=1}^{K} p(z_n|X_n, \mathbf{w}_p, \beta_p) \left(X_n^T \tilde{T}_n(z_n) \right) + \frac{\Sigma_w^{-1}}{\beta}\mu_w
\end{aligned}
$$

Where we obtain the updated value $\mathbf{w}$ by setting the above equal to zero and solving for $\mathbf{w}$:

$$
\begin{aligned}
\mathbf{w} &= \left(\sum_{n=1}^{2N}\sum_{z_n=1}^{K} p(z_n|X_n,\mathbf{w}_p,\beta_p)X_n^T X_n + \frac{\Sigma_w^{-1}}{\beta}\right)^{-1} \\
&\quad \left(\sum_{n=1}^{2N}\sum_{z_n=1}^{K} p(z_n|X_n,\mathbf{w}_p,\beta_p)X_n^T \tilde{T}_n(z_n) + \frac{\Sigma_w^{-1}}{\beta}\mu_w\right),
\end{aligned}
$$

by using:

$$
\sum_{z_n=1}^{K} p(z_n|X_n,\mathbf{w}_p,\beta_p) = 1,
$$

we can simplify the update for $\mathbf{w}$:

$$
\begin{aligned}
\mathbf{w} &= \left(X^T X + \frac{\Sigma_w^{-1}}{\beta}\right)^{-1} \\
&\quad \left(\sum_{n=1}^{2N}\sum_{z_n=1}^{K} p(z_n|X_n,\mathbf{w}_p,\beta_p)X_n^T \tilde{T}_n(z_n) + \frac{\Sigma_w^{-1}}{\beta}\mu_w\right).
\end{aligned}
$$

Which shows us that we only need $2NK$ different evaluations of some z. This in contrast to K^{2N} different values for $\mathbf{z}$ in Equation: 2. However rewriting the above as a sum over $\mathbf{z}$ shows us that $\mathbf{w}$ is a linear combination of linear classifiers:

$$
\mathbf{w} = \sum_{\mathbf{z}} p(\mathbf{z}|X,\mathbf{w}_p,\beta_p)\left(X^T X + \frac{\Sigma_w^{-1}}{\beta}\right)^{-1}\left(X^T \tilde{T}(\mathbf{z}) + \frac{\Sigma_w^{-1}}{\beta}\mu_w\right),
$$

where the combination weights are the conditional probabilities that the specific $\mathbf{z}$ is correct given the previous parameters and the labels used for training are the ones induced by $\mathbf{z}$.

2.2 Updating β

Because β does not occur in $\log p(\mathbf{w}|\mu_w,\Sigma_w)$, we only have to take the derivative of Equation: 2 and we immediately apply the result from Equation: 5:

$$
\begin{aligned}
& \frac{d}{d\beta}\sum_{\mathbf{z}} p(\mathbf{z}|X,\mathbf{w}_p,\beta_p)\log p(X,\mathbf{z}|\mathbf{w},\beta) \\
=& \sum_{n=1}^{2N}\sum_{z_n=1}^{K} p(z_n|X_n,\mathbf{w}_p,\beta_p)\frac{d}{d\beta}\log p(X_n|\tilde{T}_n(z_n),\mathbf{w},\beta).
\end{aligned}
$$

We obtain the value for $\frac{d}{d\beta}\log p(X_n|\tilde{T}_n(z_n),\mathbf{w},\beta)$ as follows:

$$\begin{aligned}
&\frac{d}{d\beta}\log p(X_n|z_n,\mathbf{w},\beta)\\
&=\sum_{r=1}^{R_n}\sum_{k=1}^{K}\frac{d}{d\beta}\log p(\mathbf{x}_{n,r,k}|\tilde{t}_{n,r,k}(z_n),\mathbf{w},\beta)\\
&=\sum_{r=1}^{R_n}\sum_{k=1}^{K}\frac{d}{d\beta}\log\mathcal{N}(\mathbf{x}_{n,r,k}\mathbf{w}|\tilde{t}_{n,r,k}(z_n),\beta^{-1})\\
&=\sum_{r=1}^{R_n}\sum_{k=1}^{K}\frac{d}{d\beta}\log\left(\frac{\beta}{2\pi}\right)^{\frac{1}{2}}\exp\left(-\frac{\beta}{2}(\mathbf{x}_{n,r,k}\mathbf{w}-\tilde{t}_{n,r,k}(z_n))^2\right)\\
&=\sum_{r=1}^{R_n}\sum_{k=1}^{K}\frac{1}{2}\frac{d}{d\beta}\log\beta-\frac{d}{d\beta}\frac{\beta}{2}(\mathbf{x}_{n,r,k}\mathbf{w}-\tilde{t}_{n,r,k}(z_n))^2\\
&=\frac{1}{2}\sum_{r=1}^{R_n}\sum_{k=1}^{K}\frac{1}{\beta}-(\mathbf{x}_{n,r,k}\mathbf{w}-\tilde{t}_{n,r,k}(z_n))^2.
\end{aligned}$$

Plugging this into the previous equation gives us:

$$\begin{aligned}
&\frac{d}{d\beta}\sum_{\mathbf{z}}p(\mathbf{z}|X,\mathbf{w}_p,\beta_p)\log p(X,\mathbf{z}|\mathbf{w},\beta)\\
=&\sum_{n=1}^{2N}\sum_{z_n=1}^{K}p(z_n|X_n,\mathbf{w}_p,\beta_p)\frac{1}{2}\sum_{r=1}^{R_n}\sum_{k=1}^{K}\left(\frac{1}{\beta}-(\mathbf{x}_{n,r,k}\mathbf{w}-\tilde{t}_{n,r,k}(z_n))^2\right).
\end{aligned}$$

This can then be solved for β after setting it equal to 0. The resulting equation is:

$$\beta^{-1} = <\sum_{z_n=1}^{K}p(z_n|X_n,\mathbf{w}_p,\beta_p)(\mathbf{x}_{n,r,k}\mathbf{w}-\tilde{t}_{n,r,k}(z_n))^2>_{n,r,k}$$

2.3 An update for α

When $\mu_w=\mathbf{0}$ and $\Sigma_W=\alpha^{-1}I$ it is also possible to optimize α. To optimize α, we only have to consider $\log p(\mathbf{w}|\alpha)$:

$$\begin{aligned}
&\frac{d}{d\alpha}\log\mathcal{N}(\mathbf{w}|\mathbf{0},\alpha^{-1}I)\\
&=\frac{d}{d\alpha}\left(\log\frac{1}{|\alpha^{-1}I|^{\frac{1}{2}}}-\frac{\alpha}{2}\mathbf{w}^T\mathbf{w}\right)\\
&=\frac{D}{2}\frac{d}{d\alpha}\log\alpha-\frac{1}{2}\mathbf{w}^T\mathbf{w}\\
&=\frac{D}{2}\frac{1}{\alpha}-\frac{1}{2}\mathbf{w}^T\mathbf{w}.
\end{aligned}$$

From which we obtain the final update

$$\alpha = \frac{D}{\mathbf{w}^T \mathbf{w}}.$$

In this case the bias term is also regularized. This might be a bad idea but we had much more problems with α going to infinity when the bias was left out of the optimization. The prior distribution on the labels forces the bias in the direction of -1. When the bias is not regularized, then there is nothing forcing the model to obtain a good fit. This results in a weight vector containing all zeros and a single -1 for the bias.
