# Supplementary material for: A Bayesian Model for Exploiting Application Constraints to Enable Unsupervised Training of a P300-based BCI
Source: PLoS One. 2012 Apr 4;7(4):e33758. doi: 10.1371/journal.pone.0033758 (PMC3319551; doi:10.1371/journal.pone.0033758)
Supplement: Document S2 — Subject Specific Results on the Akimpech Dataset. This file contains the subject specific results for all experiments performed on the Akimpech Dataset. (PDF) [file pone.0033758.s002.pdf]

S2: Subject Specific Results on the Akimpech Dataset
Supplementary Material for
A Bayesian Model for Exploiting Application Constraints to Enable Unsupervised Training of a P300 based BCI

.
.
.

Table 1: **Akimpech Spelling Accuracies using 5 repetitions**

	OFF-US	OFF-US-T	ON-US-T	OA-US-T	RE-OA-US-T	OA-US
ACS	78.4 (1.66)	77.9 (2.22)	78.9 (0.00)	78.9 (0.00)	78.9 (0.00)	59.5 (3.55)
ASG	90.5 (0.00)	90.5 (0.00)	90.5 (0.00)	90.5 (0.00)	90.5 (0.00)	76.2 (0.00)
ASR	96.2 (0.00)	96.2 (0.00)	96.2 (0.00)	96.2 (0.00)	96.2 (0.00)	93.5 (1.86)
CLL	88.5 (0.00)	80.8 (0.00)	84.6 (0.00)	76.9 (0.00)	80.8 (0.00)	23.1 (0.00)
DCM	100.0 (0.00)	100.0 (0.00)	100.0 (0.00)	100.0 (0.00)	100.0 (0.00)	85.9 (1.09)
DLP	69.6 (0.00)	78.3 (0.00)	73.9 (0.00)	73.9 (0.00)	78.3 (0.00)	47.8 (0.00)
DMA	72.9 (3.04)	70.6 (0.00)	76.5 (0.00)	76.5 (0.00)	76.5 (0.00)	24.1 (1.86)
ELC	95.7 (0.00)	95.7 (0.00)	95.7 (0.00)	91.3 (0.00)	95.7 (0.00)	91.3 (0.00)
GCE	77.9 (2.22)	78.9 (0.00)	63.2 (0.00)	78.9 (0.00)	78.9 (0.00)	43.7 (2.54)
ICE	58.3 (0.00)	58.3 (0.00)	58.3 (0.00)	54.2 (0.00)	58.3 (0.00)	25.0 (0.00)
IZH	88.5 (0.00)	92.3 (0.00)	76.9 (0.00)	88.5 (0.00)	92.3 (0.00)	46.2 (0.00)
JCR	83.3 (0.00)	83.3 (0.00)	77.8 (0.00)	88.9 (0.00)	83.3 (0.00)	25.0 (2.93)
JLD	94.1 (0.00)	100.0 (0.00)	100.0 (0.00)	100.0 (0.00)	100.0 (0.00)	82.4 (4.80)
JMR	85.7 (0.00)	95.2 (0.00)	95.2 (0.00)	95.2 (0.00)	95.2 (0.00)	43.3 (1.51)
JSC	72.1 (19.23)	68.4 (0.00)	68.4 (0.00)	68.4 (0.00)	68.4 (0.00)	36.8 (4.30)
JST	100.0 (0.00)	100.0 (0.00)	100.0 (0.00)	100.0 (0.00)	100.0 (0.00)	91.1 (2.87)
LAC	100.0 (0.00)	100.0 (0.00)	100.0 (0.00)	100.0 (0.00)	100.0 (0.00)	91.6 (2.72)
LAG	97.1 (1.51)	92.9 (0.00)	89.3 (0.00)	96.4 (0.00)	92.9 (0.00)	71.4 (0.00)
LGP	100.0 (0.00)	96.0 (0.00)	96.0 (0.00)	96.0 (0.00)	96.0 (0.00)	88.0 (0.00)
PGA	95.8 (0.00)	100.0 (0.00)	100.0 (0.00)	95.8 (0.00)	100.0 (0.00)	91.7 (0.00)
WFG	82.8 (0.00)	93.1 (0.00)	93.1 (0.00)	93.1 (0.00)	93.1 (0.00)	49.3 (4.00)
XCL	100.0 (0.00)	94.1 (0.00)	94.1 (0.00)	94.1 (0.00)	94.1 (0.00)	58.8 (0.00)

Percentage of correctly predicted characters averaged out over subjects from the Akimpech dataset. The values in braces are the standard deviation over the different initializations.

Table 2: **Akimpech Spelling Accuracies using 10 repetitions**

	OFF-US	OFF-US-T	ON-US-T	OA-US-T	RE-OA-US-T	OA-US
ACS	100.0 (0.00)	94.7 (0.00)	84.2 (0.00)	94.7 (0.00)	100.0 (0.00)	83.7 (5.79)
ASG	90.5 (0.00)	90.5 (0.00)	90.5 (0.00)	90.5 (0.00)	90.5 (0.00)	74.3 (4.02)
ASR	100.0 (0.00)	100.0 (0.00)	100.0 (0.00)	100.0 (0.00)	100.0 (0.00)	96.9 (2.43)
CLL	96.2 (0.00)	96.2 (0.00)	96.2 (0.00)	96.2 (0.00)	96.2 (0.00)	90.4 (2.72)
DCM	100.0 (0.00)	100.0 (0.00)	100.0 (0.00)	100.0 (0.00)	100.0 (0.00)	96.6 (3.25)
DLP	95.7 (0.00)	91.3 (0.00)	82.6 (0.00)	91.3 (0.00)	91.3 (0.00)	79.6 (5.44)
DMA	88.2 (0.00)	94.1 (0.00)	88.2 (0.00)	94.1 (0.00)	94.1 (0.00)	60.0 (2.48)
ELC	100.0 (0.00)	100.0 (0.00)	100.0 (0.00)	100.0 (0.00)	100.0 (0.00)	98.7 (2.93)
GCE	84.2 (0.00)	84.2 (0.00)	94.7 (0.00)	89.5 (0.00)	84.2 (0.00)	47.9 (11.49)
ICE	87.5 (0.00)	91.7 (0.00)	95.8 (0.00)	95.8 (0.00)	91.7 (0.00)	58.3 (0.00)
IZH	100.0 (0.00)	100.0 (0.00)	96.2 (0.00)	100.0 (0.00)	100.0 (0.00)	91.9 (2.84)
JCR	100.0 (0.00)	100.0 (0.00)	94.4 (0.00)	100.0 (0.00)	100.0 (0.00)	88.9 (0.00)
JLD	100.0 (0.00)	100.0 (0.00)	100.0 (0.00)	100.0 (0.00)	100.0 (0.00)	97.6 (3.04)
JMR	100.0 (0.00)	100.0 (0.00)	100.0 (0.00)	100.0 (0.00)	100.0 (0.00)	82.9 (2.46)
JSC	89.5 (0.00)	84.2 (0.00)	84.2 (0.00)	84.2 (0.00)	84.2 (0.00)	66.8 (4.99)
JST	100.0 (0.00)	100.0 (0.00)	100.0 (0.00)	100.0 (0.00)	100.0 (0.00)	100.0 (0.00)
LAC	100.0 (0.00)	100.0 (0.00)	100.0 (0.00)	100.0 (0.00)	100.0 (0.00)	93.7 (2.22)
LAG	100.0 (0.00)	100.0 (0.00)	100.0 (0.00)	100.0 (0.00)	100.0 (0.00)	90.4 (2.41)
LGP	100.0 (0.00)	96.0 (0.00)	96.0 (0.00)	96.0 (0.00)	96.0 (0.00)	95.6 (2.95)
PGA	100.0 (0.00)	100.0 (0.00)	100.0 (0.00)	100.0 (0.00)	100.0 (0.00)	88.3 (2.64)
WFG	100.0 (0.00)	100.0 (0.00)	100.0 (0.00)	100.0 (0.00)	100.0 (0.00)	96.9 (1.09)
XCL	100.0 (0.00)	100.0 (0.00)	100.0 (0.00)	100.0 (0.00)	100.0 (0.00)	82.9 (1.86)

Percentage of correctly predicted characters averaged out over subjects from the Akimpech dataset. The values in braces are the standard deviation over the different initializations.

Table 3: **Akimpech Spelling Accuracies using 15 repetitions**

	OFF-US	OFF-US-T	ON-US-T	OA-US-T	RE-OA-US-T	OA-US
ACS	94.7 (0.00)	94.7 (0.00)	89.5 (0.00)	94.7 (0.00)	94.7 (0.00)	90.0 (5.23)
ASG	95.2 (0.00)	95.2 (0.00)	100.0 (0.00)	95.2 (0.00)	95.2 (0.00)	94.8 (1.51)
ASR	100.0 (0.00)	100.0 (0.00)	100.0 (0.00)	100.0 (0.00)	100.0 (0.00)	100.0 (0.00)
CLL	100.0 (0.00)	100.0 (0.00)	100.0 (0.00)	100.0 (0.00)	100.0 (0.00)	93.1 (1.62)
DCM	100.0 (0.00)	100.0 (0.00)	100.0 (0.00)	100.0 (0.00)	100.0 (0.00)	99.7 (1.09)
DLP	100.0 (0.00)	100.0 (0.00)	91.3 (0.00)	100.0 (0.00)	100.0 (0.00)	87.0 (4.10)
DMA	94.1 (0.00)	94.1 (0.00)	94.1 (0.00)	94.1 (0.00)	94.1 (0.00)	86.5 (2.84)
ELC	100.0 (0.00)	100.0 (0.00)	100.0 (0.00)	100.0 (0.00)	100.0 (0.00)	97.0 (3.58)
GCE	89.5 (0.00)	89.5 (0.00)	89.5 (0.00)	89.5 (0.00)	89.5 (0.00)	75.3 (4.99)
ICE	100.0 (0.00)	100.0 (0.00)	100.0 (0.00)	100.0 (0.00)	100.0 (0.00)	90.0 (2.91)
IZH	100.0 (0.00)	100.0 (0.00)	100.0 (0.00)	100.0 (0.00)	100.0 (0.00)	98.5 (1.99)
JCR	100.0 (0.00)	100.0 (0.00)	100.0 (0.00)	100.0 (0.00)	100.0 (0.00)	92.2 (3.88)
JLD	100.0 (0.00)	100.0 (0.00)	100.0 (0.00)	100.0 (0.00)	100.0 (0.00)	98.8 (2.48)
JMR	100.0 (0.00)	100.0 (0.00)	100.0 (0.00)	100.0 (0.00)	100.0 (0.00)	93.8 (2.30)
JSC	100.0 (0.00)	100.0 (0.00)	94.7 (0.00)	89.5 (0.00)	100.0 (0.00)	83.7 (2.99)
JST	100.0 (0.00)	100.0 (0.00)	100.0 (0.00)	100.0 (0.00)	100.0 (0.00)	97.8 (2.87)
LAC	100.0 (0.00)	100.0 (0.00)	100.0 (0.00)	100.0 (0.00)	100.0 (0.00)	94.2 (4.61)
LAG	100.0 (0.00)	100.0 (0.00)	100.0 (0.00)	100.0 (0.00)	100.0 (0.00)	97.5 (2.41)
LGP	100.0 (0.00)	100.0 (0.00)	96.0 (0.00)	100.0 (0.00)	100.0 (0.00)	93.2 (5.01)
PGA	100.0 (0.00)	100.0 (0.00)	100.0 (0.00)	100.0 (0.00)	100.0 (0.00)	91.2 (2.37)
WFG	100.0 (0.00)	100.0 (0.00)	96.6 (0.00)	100.0 (0.00)	100.0 (0.00)	94.5 (1.78)
XCL	100.0 (0.00)	100.0 (0.00)	100.0 (0.00)	100.0 (0.00)	100.0 (0.00)	98.8 (2.48)

Percentage of correctly predicted characters averaged out over subjects from the Akimpech dataset. The values in braces are the standard deviation over the different initializations.
